# Efficacy of albumin-bound paclitaxel combined with nedaplatin in neoadjuvant therapy for esophageal squamous cell carcinoma: A single-center retrospective observational study

**DOI:** 10.1097/MD.0000000000033157

**Published:** 2023-03-03

**Authors:** Jiakuan Chen, Jianfei Zhu, Yan Zhang, Wenchen Wang, Yanmin Xia, Jinbo Zhao, Tao Jiang

**Affiliations:** a Department of Thoracic Surgery, Fourth Military Medical University, Xi’an, China; b Department of Thoracic Surgery, Shaanxi Provincial People’s Hospital, Xi’an, China; c Department of Pathology, Fourth Military Medical University, Xi’an, China.

**Keywords:** albumin-bound paclitaxel, esophageal squamous cell carcinoma, nedaplatin, neoadjuvant chemotherapy, pathological complete response (pCR), tumor regression grade (TRG)

## Abstract

This study was designed to observe the efficacy and safety of albumin-bound paclitaxel plus nedaplatin as neoadjuvant therapy in patients with esophageal squamous cell carcinoma (ESCC). From April 2019 to Dec 2020, patients with ESCC who underwent Mckeown surgery at our center were analyzed retrospectively. All patient received 2 to 3 cycles of albumin-bound paclitaxel combined with nedaplatin before surgery, tumor regression grade (TRG) and American National Cancer Institute Common Toxicity Criteria version 5.0 were used to evaluate its efficacy and safety. TRG grades from TRG 2 to TRG 5are considered effective in chemotherapy, TRG 1 stands for pathological complete response (pCR). A total of 41 patients were included in this study. All patients achieved R0 resection. According to the TRG classification, the number of patients assessed for TRG 1-TRG 5 were: 7 cases, 12 cases, 3 case, 12 cases and 7 cases. Its objective response rate and pCR were 82.9% (34/41) and 17.1% (7/41), respectively. We found that hematological toxicity is the most common adverse events of this regimen, with an incidence of 24.4%, followed by digestive tract reactions, with an incidence of 17.1%. Hair loss, neurotoxicity and hepatological disorder are the others, their incidence was 12.2%, 7.3%, and 2.4%; and chemotherapy related deaths were no found. Notably, 7 patients achieved pCR without recurrence or death. Survival analysis showed that patients with pCR may have longer disease-free survival (*P* = .085) and overall survival (*P* = .273), although the difference was not statistically significant. As neoadjuvant therapy for patients with ESCC, albumin-bound paclitaxel combined with nedaplatin has a higher pCR rate and less side effects. It is a reliable choice for ESCC patients as neoadjuvant therapy.

## 1. Introduction

Radical resection is the standard treatment for early stage of esophageal squamous cell carcinoma (ESCC), but the long term outcomes is still not satisfactory. The main factors affecting the prognosis were postoperative tumor residual and postoperative recurrence and metastasis.^[1–3]^ Neoadjuvant chemotherapy (NCT) and neoadjuvant chemoradiotherapy (NCRT) can considerably reduce the postoperative recurrence and metastasis of ESCC, subsequently, improve the long term survival rate. In particular, neoadjuvant chemoradiotherapy data from the CROSS trial^[4]^ and NEOCRTEC5010 trial^[5]^ established NCRT as the standard of care in patients with advanced ESCC. However, the more postoperative complications and higher postoperative mortality brought by this mode of treatment cannot be ignored.^[6]^ Therefore, more effective and less toxic neoadjuvant therapy regimens are being sought to improve the clinical outcome of ESCC patients.

The selection of neoadjuvant chemotherapy is controversial. JCOG9907 trial^[7]^ confirmed preoperative chemotherapy with cisplatin combination with 5-fuorouracil as the standard treatment for patients with advanced ESCC. JCOG1109 trial^[8]^ further showed that DCF regimen with docetaxel addition may be better than combination with 5-fuorouracil regimen. However, the toxicity of this regimen, especially hematotoxicity, is of concern.

Previous studies^[9,10]^ have confirmed that albumin-bound paclitaxel has shown good clinical efficacy in the treatment of advanced ESCC, but the role of this regimen in the neoadjuvant treatment of operable ESCC currently lacks clinical data support. Therefore, in this study, we used tumor regression grade (TRG) grading to evaluate the efficacy and safety of albumin-bound paclitaxel combined with nedaplatin in neoadjuvant therapy for ESCC.

## 2. Material and Methods

### 2.1. Study design

A retrospective analysis was performed on thoracic ESCC patients who received surgery in Tangdu Hospital affiliated to Fourth Military Medical University from April 2019 to December 2020. The 8th version of American Joint Committee on Cancer was used for preoperative and postoperative staging of the patients.^[11]^ Inclusion criteria as follows: Thoracic ESCC received Mckeown resection; Lymph node dissection ≥ 2.0 field; Preoperative albumin-binding paclitaxel combined with nedaplatin chemotherapy for 2 to 3 cycles, and; Postoperative TRG analysis was performed.

### 2.2. Neoadjuvant chemotherapy regimen

All patients received 2 to 3 cycles of chemotherapy, albumin-binding paclitaxel administered at dose of 130 mg/m^2^, day 1 and day 8; nedaplatin 50 mg/m^2^, at day 1 and day 2; every 3 weeks.^[12]^ Mckeown surgery was performed 3 to 4 weeks after the end of the last chemotherapy. After each chemotherapy, the adverse events of chemotherapy were closely observed and treated accordingly.

### 2.3. Evaluation of chemotherapy efficacy and adverse events

All surgical specimens were treated with standard pathological procedures to assess TRG. Briefly, gross specimens were fixed with 10% neutral buffer formalin immediately after collection, followed by paraffin embedding. Continuous sections 4 μm thick were stained with H&E, as described in our previous study.^[13]^ The efficacy of neoadjuvant chemotherapy was evaluated by TRG, with reference to the Mandard TRG criteria in esophageal cancer.^[14]^ The efficacy of neoadjuvant chemotherapy for ESCC was divided into 5 grades: TRG1-5, TRG 1: complete fibrosis with no evidence of residual tumor, complete regression, TRG 2: massive fibrosis with scattered tumor cells, TRG 3: coexistence of fibrosis and residual tumor, predominant fibrosis, TRG 4: fibrosis and residual tumor coexist, dominated by tumor, TRG 5: extensive residual tumor, no evidence of regression. TRG evaluation as TRG 1 to TRG 4 was considered as objective response rate. TRG assessment were independently assessed by 2 experienced pathologists (Dr Yan Zhang and Dr Li Gong). If there was any difference in judgment, and the major disagreement between the 2 doctors was checked by a third reviewer. Adverse events for chemotherapy regimens were applicable to the National Cancer Institute Common Toxicity Criteria 5.0, which could be applied to grades 1 to 5 according to severity.^[15]^

### 2.4. Statistical analysis

Qualitative data were compared with the Mann–Whitney *U* test and χ^2^test, and categorical variables were compared by Fisher exact test. Disease-free survival (DFS) and overall survival (OS) curves were generated using a log rank. All statistics were analyzed using SPSS 22.0 (IBM, New York, NY) and Stata 14.0 (Texas, USA), and *P* < .05 was considered statistically significant.

## 3. Results

### 3.1. Patient characteristics

A total of 41 patients were included in this study, including 34 males and 7 females, with median age was 61 years (range: 38–77 years); 10 cases were located in the upper, 30 cases were located in the middle, and 1 case located in the lower. Two cases were well differentiated (G1), 29 cases were moderately differentiated (G2), and 10 cases were poorly differentiated (G3). Twenty-three patients belong to N negative and 18 patients belong to N positive before operation. Patients in cT3 stage (28 cases) and cT4 stage (8 cases) accounted for 87.8% of all cases. According to the American Joint Committee on Cancer stage of the 8th edition, there were 1 case in stage I, 19 cases in stage II, and 21 cases in stage III (Table 1).

**Table 1:** Clinical characteristics of patients with operable ESCC.

Variables	N	%
Gender		
Male	34	82.9
Female	7	17.1
Age		
< 61 yr	16	39.0
≥61 yr	25	61.0
Smoking status		
Non smoker	8	19.5
Smoker	33	80.5
Drinking status		
Non drinker	20	48.8
Drinker	21	51.2
Differentiation		
G1	2	4.9
G2	29	70.7
G3	10	24.4
Family history of tumour		
Without	30	73.2
With	11	26.8
Tumour location		
Upper	10	24.4
Middle	30	73.2
Lower	1	2.4
Clinical *T* stage		
*T*1	1	2.4
*T*2	4	9.8
*T*3	28	68.3
*T*4	8	19.5
Clinical N stage		
N negative	23	56.1
N positive	18	43.9
Clinical TNM Stage		
Incubation stage	1	2.4
Incubation stage	19	46.3
Incubation stage	21	51.3

ESCC = esophageal squamous cell carcinoma.

### 3.2. Efficacy evaluation of chemotherapy

All patients received Mckeown surgery, and all of them achieving R0 resection (100%). According to the Mandard TRG criteria, 17.1% (7 cases) of patients were assessed as TRG 5 pathological complete response (pCR), 29.3% (12 cases) as TRG 4, 7.2% (3 cases) as TRG 3, 29.3% (12 cases) as TRG 2, and 17.1% (7 cases) as TRG 1. There was no clear correlation between clinicopathological features and TRG grading after chemotherapy (Table 2). The pathological characteristics of pCR patients was showed in Table 3.

**Table 2:** Correlation between TRG and clinicopathological characteristics after neoadjuvant for patients with ESCC.

Variables	TRG					*P* value
	TRG 5	TRG 4	TRG 3	TRG 2	TRG 1	
Gender						.859
Male	6	10	3	10	5	
Female	1	2	0	2	2	
Age						.437
<61 yr	5	4	1	4	2	
≥61 yr	2	8	2	8	5	
Smoking status						.825
Non smoker	1	3	0	2	2	
Smoker	6	9	3	10	5	
Drinking status						.463
Non drinker	4	6	0	7	3	
Drinker	3	6	3	5	4	
Differentiation						.412
G1	1	1	0	0	0	
G2	4	6	3	11	5	
G3	2	5	0	1	2	
Family history of tumour						.635
Without	6	7	2	9	6	
With	1	5	1	3	1	
Tumour location						.678
Upper	1	3	1	3	2	
Middle	6	9	2	9	4	
Lower	0	0	0	0	1	
Clinical *T* stage						.709
*T*1	0	1	0	0	0	
*T*2	0	3	0	1	0	
*T*3	5	7	2	9	5	
*T*4	2	1	1	2	2	
Clinical N stage						.764
N negative	3	7	1	7	5	
N positive	4	5	2	5	2	
Clinical TNM stage						.708
I stage	0	1	0	0	0	
II stage	2	5	1	6	5	
III stage	5	6	2	6	2	

ESCC = esophageal squamous cell carcinoma, TRG = tumor regression grade.

**Table 3:** Characteristics of patients achieved pCR after NCT.

No	Gender	Age	Differentiation	Tumor location	cT stage	cN stage	TNM stage
1	Male	65	G2	Upper	*T*3	N negative	II stage
2	Male	58	G2	Middle	*T*3	N negative	II stage
3	Male	77	G3	Middle	*T*4	N positive	III stage
4	Male	46	G2	Lower	*T*4	N positive	III stage
5	Female	69	G3	Upper	*T*3	N negative	II stage
6	Female	62	G2	Middle	*T*3	N negative	II stage
7	Male	69	G2	Middle	*T*3	N negative	II stage

NCT = neoadjuvant chemotherapy, pCR = pathological complete response.

**Figure 1.**
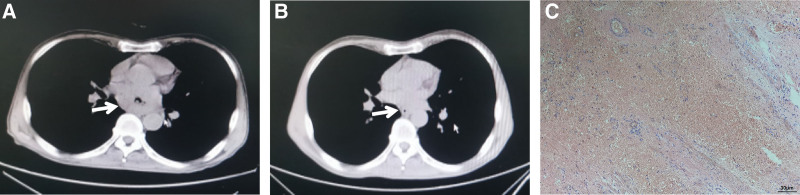
Radiological features and TRG evaluation of patient with the ESCC before and after NCT. (A) The imaging manifestations of ESCC before chemotherapy, (B) the primary tumor shrinked significantly after treatment, and (C) TRG confirmed that the tumor has achieved a pCR. ESCC = esophageal squamous cell carcinoma, NCT = neoadjuvant chemotherapy, pCR = pathological complete response, TRG = tumor regression grade.

**Figure 2.**
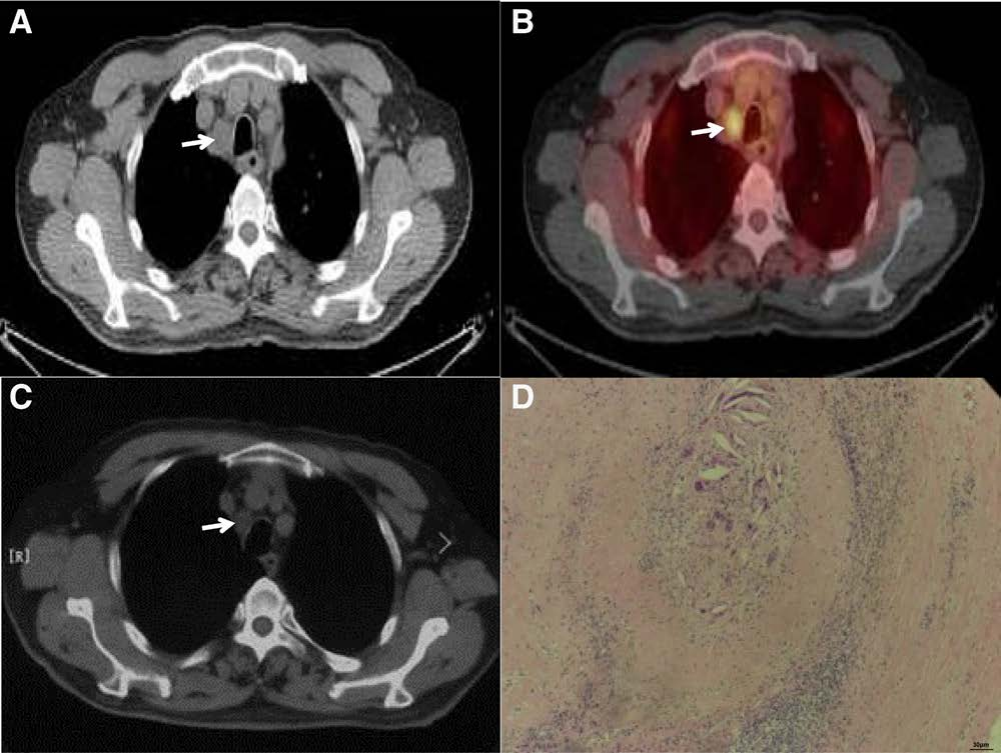
Efficacy evaluation of NCT for metastatic lymph nodes of ESCC. (A) CT confirmed that the lymph nodes adjacent to the right recurrent laryngeal nerve were metastatic lymph nodes (short diameter: 1.7 cm), (B) PET/CT confirmed that this lymph node was a metastatic lymph node, (C) right recurrent laryngeal parastinal lymph node reduction after chemotherapy (short diameter: 1.0 cm), and (D) TRG confirmed that the metastatic lymph nodes has achieved a pCR. ESCC = esophageal squamous cell carcinoma, NCT = neoadjuvant chemotherapy, pCR = pathological complete response, TRG = tumor regression grade.

By comparing the downstaging effect before and after treatment, we analyzed the effect of this regimen on downstaging in primary tumor (T stage) and on metastatic lymph nodes (N stage), respectively. For the T stage, 51.2% (21/41) of patients were down staged after treatment, it was showed in Figure 1. Of the 18 patients with preoperative assessment of lymph node metastasis, 2 patients had negative lymph node status after surgery, suggesting that this regimen is equally effective for metastatic lymph nodes (Fig. 2).

### 3.3. Adverse events evaluation

After chemotherapy, all patients were evaluated for adverse events according to National Cancer Institute Common Toxicity Criteria 5.0. Among them, hematologic toxicity was the most common toxicity and side effects, with an incidence rate of 24.4%; followed by gastrointestinal reactions, with an incidence rate of 17.1%; hair loss, neurological toxicity and liver function damage were the common side effects of chemotherapy, the incidence was 12.2%, 7.3%, and 2.4%. Adverse events of grade 4 and above was not observed in this study, in Table 4.

**Table 4:** Adverse events of albumin-bound paclitaxel combined with nedaplatin.

Type	Adverse reaction				
	Grade 1	Grade 2	Grade 3	Grade 4	Grade 5
Blood toxicity	3	4	3	0	0
Peripheral sensory neuropathy	2	1	0	0	0
Hepatorenal disorder	1	0	0	0	0
Digestive tract reaction	5	2	0	0	0
Allergic reaction	0	0	0	0	0
Hair loss	2	3	0	0	0

### 3.4. Survival analysis

**Figure 3.**
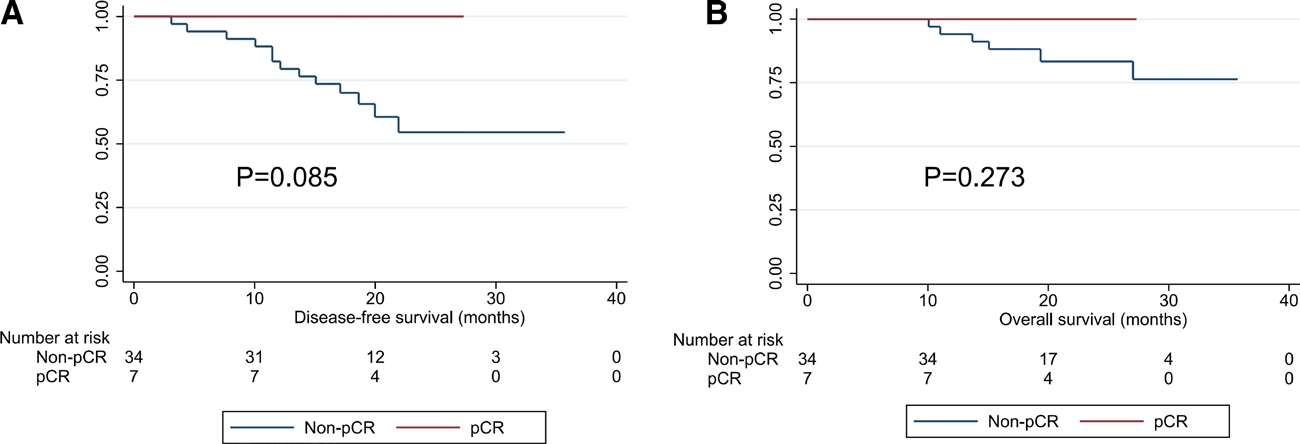
Survival curves of ESCC with different status of pCR. (A) DFS of ESCC according to the status of pCR and (B) OS of ESCC according to the status of pCR. DFS = disease-free survival, ESCC = esophageal squamous cell carcinoma, OS = overall survival, pCR = pathological complete response.

As of March 16, 2022, with a median follow up of 23.4 months, 10 patients developed recurrence and metastasis after surgery, and 6 patients died. Notably, 7 patients achieved pCR without recurrence or death. Survival analysis showed that patients with pCR may have longer DFS (*P* = .085) and OS (*P* = .273), it was showed in Figure 3, although the difference was not statistically significant.

## 4. Discussion

For patients with operable ESCC, adjuvant therapy, especially NCRT, is the recommended mode of treatment according to various clinical guidelines. A prospective study confirmed that NCRT was effective, in this study, a total of 451 patients were included, including 224 patients receiving NCRT and 227 patients receiving surgery alone. They found that NCRT significantly reduced the residue positive rate (98.4% vs 91.2%, *P* = .002), and the postoperative pCR rate reached 43.2%. Survival analysis showed that, compared with patients receiving surgical treatment alone, patients received NCRT had longer median DFS (*P* = .001) and OS (*P* = .025).^[5]^ It was the largest sample size study on the prolongation of survival in patients with ESCC by NCRT. However, neoadjuvant concurrent chemoradiotherapy also faces the following problems: First, NCRT increases the risk of surgery and the treatment cost of patients.^[16,17]^ Second, the requirements of radiotherapy technology and equipment for medical institutions make NCRT could not be carried out in an all-round way, and the requirements for the whole-process management of ESCC patients are relatively strict. Therefore, to find a reasonable and reliable preoperative NCT regimen is a research hotspot in ESCC. This study retrospectively analyzed the effect of albumin-bound paclitaxel combined with nedaplatin in neoadjuvant therapy for ESCC, and the results showed that 82.9% of patients were effective for chemotherapy, suggesting that this regimen is one of the effective neoadjuvant therapies for ESCC.

There is no uniform clinical standard for NCT for ESCC. A study by Japanese researchers called JCOG9907,^[7]^ a total of 330 patients were included, and the chemotherapy regimen was 5-fluorouracil plus cisplatin. Patients were randomly divided into neoadjuvant chemotherapy group and adjuvant chemotherapy group. R0 resection rate of NCT patients was 72%, while that of postoperative chemotherapy group was 57%. In addition, NCT could extended the 5-year survival rate of patients by 55% compared with 43% (RR: 0.73, 95% CI: 0.54–0.99, *P* = .04). Different chemotherapy regimens had different pCR rates, ranging from 2% to 17%.^[18]^ A study of paclitaxel combined with cisplatin/nedaplatin in NCT was confirmed, the pCR rate of the scheme could reach 20.5%, and the survival time of the patients was prolonged, *P* = .049.^[19]^ In our study, all patients receiving the albumin-binding paclitaxel combined with nedaplatin regimen achieved R0 resection, and 17.1% of the patients arrived at pCR, suggesting that many ESCC patients might benefit from this regimen.

As a third-generation paclitaxel drug, albumin-bound paclitaxel uses nanotechnology to make albumin and paclitaxel into nanoparticles, and achieves the realization of the targeting function of chemotherapeutic drugs, and secondly, albumin-bound paclitaxel does not require solvent to dissolve, reducing the incidence of allergic reactions.^[20,21]^ These 2 characteristics make it widely used in the treatment of malignant tumors. Zhang et al^[22]^ evaluated the efficacy and safety of nab-paclitaxel combined with cisplatin and capecitabine triple-drug NCT in locally advanced ESCC. Of the 21 patients who received this regimen and ultimately underwent surgery, 8 (38.1%) achieved pCR, however, 35.5% patients had grade 3/4 chemotherapy adverse events. No chemotherapy adverse events above grade 4 were found in our study, suggesting that patients with the 2-drug regimen will have better tolerance.

The evaluation method of adjuvant therapy for ESCC is controversial. It is well known that for solid tumors, response evaluation criteria in solid tumors (RECIST) criteria are widely used to evaluate the efficacy of solid tumor therapy.^[23,24]^ As a hollow organ, the clinical *T* staging of esophageal cancer mainly depends on the depth of tumor invasion rather than the size of the tumor. Therefore, it is obviously unreasonable to use RECIST criteria to evaluate the tumor. In surgical ESCC, the TRG system was introduced to assess the effectiveness of NCT.^[25]^ Guo et al^[26]^ applied TRG evaluation system to evaluate the efficacy of NCT for ESCC, and found that the efficacy of TRG in NCT for ESCC could be objectively evaluated and the patients with ultimate survival benefit could be screened. The therapeutic effect of chemotherapy was evaluated more quantitatively, suggesting that TRG should be widely used in the efficacy evaluation of neoadjuvant therapy for ESCC. The evaluation of lymph nodes in esophageal cancer is currently a clinical challenge. Interestingly, in our study, 1 patient who was evaluated for lymph node metastasis on the right recurrent laryngeal nerve before chemotherapy, was also confirmed to be metastatic lymph nodes by PET/CT. After chemotherapy, according to RECIST criteria, the evaluation of efficacy was PR, but TRG evaluation was TRG 1, which confirmed that TRG was more objective than RECIST in evaluating the efficacy of neoadjuvant therapy for ESCC.

Recently, the results of immunological neoadjuvant therapy in esophageal cancer are exciting.^[12,27]^ An open-label, single-arm, single-center Phase 2 clinical trial of sintilimab in combination with chemotherapy (nab-paclitaxel + cisplatin) as neoadjuvant therapy for ESCC was published. A total of 30 patients were enrolled. According to the Reciest assessment, the objective response rate and disease control rate were 67% (20/30), respectively, and the disease control rate was 97% (29/30). Finally, 23 patients underwent McKeown minimally invasive radical esophagectomy. The pCR rate of primary tumors was 21.7%, and the MPR rate of primary tumors was 52.2%.^[28]^ Compared with this study, in our study, the pCR was still as high as 17.1% without the addition of immunotherapy, suggesting that this regimen is the choice of neoadjuvant therapy for ESCC.

It is undeniable that there are some limitations in this study. First, although this study has the largest sample size of this program, the sample size is still not enough for us to conduct further sub-analysis; Secondly, it is a single-arm observation study, without a control group, could not allow comparative analysis; Finally, the short follow up time limited the survival analysis in this study.

In conclusion, this study preliminarily confirmed that the albumin-binding paclitaxel combined with nedaplatin regimen has good clinical effect and tolerable toxicity in patients with operable ESCC, and is one of the alternative regiments for neoadjuvant therapy of ESCC patients.

## Author contributions

**Conceptualization:** Jiakuan Chen.

**Data curation:** Jianfei Zhu, Wenchen Wang.

**Formal analysis:** Yanmin Xia.

**Investigation:** Jinbo Zhao, Tao Jiang.

**Methodology:** Yan Zhang.
